# Study on Thermal Oxygen Aging Characteristics and Degradation Kinetics of PMR350 Resin

**DOI:** 10.3390/polym16182552

**Published:** 2024-09-10

**Authors:** Yadan Wu, Wenchen Zhao, Yang Liu, Haitao Liu, Minglong Yang, Xun Sun

**Affiliations:** Science and Technology on Advanced Ceramic Fibers and Composites Laboratory, College of Aerospace Science and Engineering, National University of Defense Technology, Changsha 410073, China; wyd815716@163.com (Y.W.); 15306319761@163.com (W.Z.); liuyang72s@163.com (Y.L.); htssunxun@163.com (X.S.)

**Keywords:** polyimide, thermal-oxidative aging, thermal degradation kinetics, micromechanical properties

## Abstract

The thermal stability and aging kinetics of polyimides have garnered significant research attention. As a newly developed class of high thermal stability polyimide, the thermal aging characteristics and degradation kinetics of phenylene-capped polyimide prepolymer (PMR350) have not yet been reported. In this article, the thermo-oxidative stability of PMR350 was investigated systematically. The thermal degradation kinetics of PMR350 resin under different atmospheres were also analyzed using the Flynn–Wall–Ozawa method, the Kissinger–Akahira–Sunose method, and the Friedman method. Thermogravimetric analysis (TGA) results revealed that the 5% thermal decomposition temperature (Td5%) of PMR350 in a nitrogen atmosphere was 29 °C higher than that in air, and the maximum thermal degradation rate was 0.0025%/°C, which is only one-seventh of that observed in air. Isothermal oxidative aging results indicated that the weight loss rate of PMR350 and the time-dependence relationship follow a first-order exponential growth function. PMR350 resin thermal decomposition reaction under air atmosphere includes one stage, with a degradation activation energy of approximately 57 kJ/mol. The reaction model g(α) fits the F_2_ model, and the integral form is given by g(α) = 1/(1 − α). In contrast, the thermal decomposition reaction under a nitrogen atmosphere consists of two stages, with degradation activation energies of 240 kJ/mol and 200 kJ/mol, respectively. The reaction models g(α) correspond to the A_2_ and D_3_ models, with the integral forms represented as g(α) = [−ln(1 − α)]^2^ and g(α) = [1 − (1 − α)^1/3^]^2^ due to the oxygen accelerating thermal degradation from multiple perspectives. Moreover, PMR350 resin maintained high hardness and modulus even after thermal aging at 350 °C for 300 h. The results indicate that the resin exhibits excellent resistance to thermal and oxygen aging. This study represents the first systematic analysis of the thermal stability characteristics of PMR350 resin, offering essential theoretical insights and data support for understanding the mechanisms of thermal stability modification in PMR350 and its engineering applications.

## 1. Introduction

The significant aerothermal effects of high-speed aircraft, such as rockets and so on, create an urgent demand for high-temperature structural materials. As one of the primary classes of high-temperature resin matrix composites, thermosetting polyimide (PI) resin matrix composites offer outstanding advantages, including excellent high-temperature stability, lightweight properties, and high strength [1,2]. Consequently, they are widely used in military and civilian engineering applications as well as in aerospace transportation vehicles. However, in recent years, the continuous increase in the flight speed of aerospace vehicles has led to a sharp rise in the temperature of the composites on the fuselage’s surface during high-speed flight. The elevated temperature and thermal stress can result in changes or even damage to the material structure, significantly affecting its mechanical properties [3]. To address the requirements for structural strength and thermal deformation control of aerospace aircraft under aerodynamic heating (high-temperature) conditions, it is essential to conduct research on high-performance resin matrix composites with high-temperature resistance [4,5,6,7].

Research on the thermal oxidation stability, processing performance, and comprehensive mechanical properties of thermosetting polyimide composite materials has received wide attention [8,9]. Various modification methods, including chemical structure modification methods, physical doping modification methods, and other approaches, have been developed to enhance thermal oxidation stability. Chemical structure modification is the most direct method to improve polyimide’s thermal oxidation stability. However, the improvement is limited based on the molecular structure, and the introduction of the carbon borane molecular backbone unit structure has been reported as the most effective modification method to enhance polyimide resin’s thermal oxidation stability [10], despite the high cost of monomer preparation. Physical doping modification methods, such as incorporating alumina [11], carbon nanotubes [12,13], graphene [14,15], inorganic compounds, and polyimide compounds, have been reported as low-cost and high-efficiency approaches to enhance heat resistance. However, the increase in thermal oxidation stability is relatively limited, making them suitable for specific scenarios with functional requirements. Despite efforts to enhance the temperature tolerance of polyimide materials, the practical engineering applications may still face limitations. For instance, oxidation at high temperatures can result in chain fracture of polyimide molecules and thermal degradation of the resin. Prolonged exposure to high-temperature environments may lead to a decline in the mechanical properties of polyimide composite resin-based materials [16]. The long-term reuse at high temperatures is a fundamental requirement for aviation structural composite materials. Currently, the aviation industry is testing resistance at 350 °C and 400 °C, which imposes more stringent demands on the long-term thermal oxygen aging ability and toughness of matrix resin. Therefore, the thermal oxidation stability (TOS) of polyimide composites has become a key index for evaluating their performance. Studying the thermo-oxidative aging process of polyimide is of great significance to understand the aging characteristics of polyimide.

Thermal oxidative degradation of thermosetting polyimide resins is very complex, involving multiple chemical reaction processes. Currently, there is no unified theoretical model established for this process. The use of thermal degradation kinetics has emerged as an effective tool for studying resin degradation in recent years, with commonly used methods including the Kissinger, Friedman, Flynn–Wall–Ozawa, and Kissinger–Akahira–Sunose methods, and others [17]. The Kissinger method [18,19] is favored in the study of the degradation behavior of polyimide. Chang et al. [20] conducted a detailed study on the thermal oxidation degradation behavior of siloxide-containing structured polyimide. Through this method, the thermal degradation mechanism can be better understood by analyzing the polyimide degradation process. Gu et al. [21] utilized the Kissinger method to investigate the kinetic parameters of the thermal degradation of aromatic polyimide, aiming to comprehensively understand the thermal degradation mechanism and establish a foundation for its high-temperature applications. The Kissinger method may not be suitable when the resin decomposition involves multiple step reactions and multiple peaks, as the temperature point corresponding to the peak needs to be selected during the calculation process. The Friedman method, a differential method commonly used to determine the kinetic parameters of thermal decomposition reactions, has been employed more frequently [22,23] in the degradation studies of phenolic resin and polyether ether ketone resin. The Kissinger–Akahira–Sunose method, similar to the Flynn–Wall–Ozawa method, is an integration method. When using these two methods to determine the thermal decomposition kinetic parameters, there is no need to understand the thermal decomposition mechanism or introduce thermal loss differential data. The two methods are often used together to verify the reliability of the calculated data [24,25]. In order to speculate the thermal degradation mechanism of the resin, people often use the Coats-Redfern method to compare the test data with the standard curve to predict the chemical reaction of the resin degradation process [26].

Phenylacetylene group-sealing end polyimide resin (PMR350) is a newly explored high-performance polyimide resin with improved temperature resistance. The analysis of its thermal properties has not been reported. To evaluate its temperature resistance, TGA and isothermal oxygen aging methods were used to test the thermal oxidative degradation dynamics based on the classical theories of Flynn–Wall–Ozawa, Kissinger–Akahira–Sunose, and Friedman, and analysis. This is the first systematic work to analyze the thermal stability characteristics of PMR350 resin, providing key theoretical and data support for understanding the thermal stability modification mechanism of PMR350 and its engineering application.

## 2. Experiment and Characterization

### 2.1. Experimental Material

Phenylene-capped polyimide prepolymer (PMR350), a brown–green liquid, was developed by the Ningbo Institute of Materials Technology and Engineering, Chinese Academy of Sciences. The specific parameters are detailed in Table 1.

### 2.2. Material Characterization

(1) TGA analysis: The STA499 F3 thermal analyzer by Netzsch (Waldkraiburg, Germany) was utilized in both nitrogen and air atmospheres. The test temperature ranged from room temperature to 800 °C, with heating rates of 3, 5, 8, and 10 °C/min. This was performed to examine the TGA curve of PMR350 polyimide resin under various heating rates.

(2) Fourier transform infrared spectroscopy (FT-IR): The Vector-22 infrared spectrometer from Bruker (Berlin, Germany) was utilized. The scanning range was 4000~400 cm^−1^ with 128 scanning times at a resolution of 4 cm^−1^, and testing was conducted on a sample powder and KBr mixed tablet.

(3) Hot oxidation experiment: The high-temperature oven of DHG-9039A of Heheng (Yibin, China) is used as a hot oxygen aging test equipment, and the gas flow rate of the oven is the fixed value of the equipment. Initially, the PMR350 polyimide resin cured sample was placed in the high-temperature oven, heated to 200 °C for 1 h, then removed and cooled to room temperature in a dryer. It was weighed using an analytical balance (accurate to 0.00001 g), and the recorded value was considered the initial weight (m_0_). Subsequently, the composite sample was placed back into the high-temperature oven, heated to 350 °C at a rate of 3 °C/min, and maintained at a constant temperature, and the time was recorded. The sample was removed and weighed every 25 h, recorded as m_tn_ (n = 25, 50, 75, 100...). The weight loss rate at that time is calculated according to Equation (1):

(1) $$\Delta m=(m_{tn}-m_{0})/m_{0}\times 100\%$$

(4) Scanning electron microscopy (SEM): The JSM-7610FPlus scanning electron microscope (Japan) was used to analyze and characterize the surface and cross-section of the resin samples before and after thermal aging.

(5) Micromechanical properties test: The hardness and modulus of the resin samples before and after thermal examination were tested using the Hysitron T1950 nanoindentation instrument manufactured by Bruker (Germany). The loading mode selects the loading force control mode and sets the maximum loading force to 10,000 mN.

## 3. Results and Discussion

### 3.1. Preparation of PMR350 Prepolymer Powder and Solidified Sample

**Figure 1:**
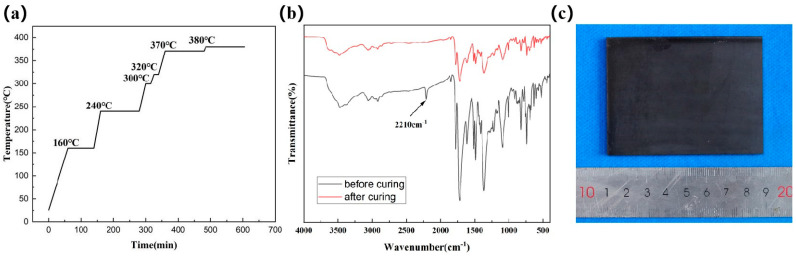
(**a**) PMR350 resin curing process; (**b**) Comparison of infrared test results before and after resin curing; (**c**) Photographs of the resin cure.

The resin prepolymer was heated from room temperature to 100 °C for 3 h for drying, and the dry product was ground to obtain prepolymer powder, while the rest was cured. The specific curing process is shown in Figure 1a. The ground powder was used for the TGA test, and the prepolymer powder and solidified powder were used for infrared testing using the KBr tablet method. The band ranges from 400 to 4000 cm^−1^, as shown in Figure 1b. As observed in the figure, the symmetric (1780 cm^−1^) and asymmetric stretching vibration characteristic peaks of the imide carbonyl group (1721 cm^−1^), and the vibration characteristic peaks of the imide ring (1370 cm^−1^, 1110 cm^−1^, and 740 cm^−1^) did not change significantly before and after the curing crosslinking reaction, indicating that the imidization degree of the resin was complete. The characteristic absorption peak of C≡C (2210 cm^−1^) of the phenylidene group basically disappeared after curing for 2 h at 380 °C and 5 MPa, indicating that the prepolymer underwent a curing crosslinking reaction and the curing process was complete, demonstrating that the hot pressing process is reliable.

Phenylene-ended polyimide PMR350 resin, cured as a brown–black bulk solid, was prepared by the Ningbo Institute of Materials Technology and Engineering, Chinese Academy of Sciences, using a molding method based on the curing process depicted in Figure 1a. The specific morphology is illustrated in Figure 1c.

### 3.2. Thermal Degradation Performance

**Figure 2:**
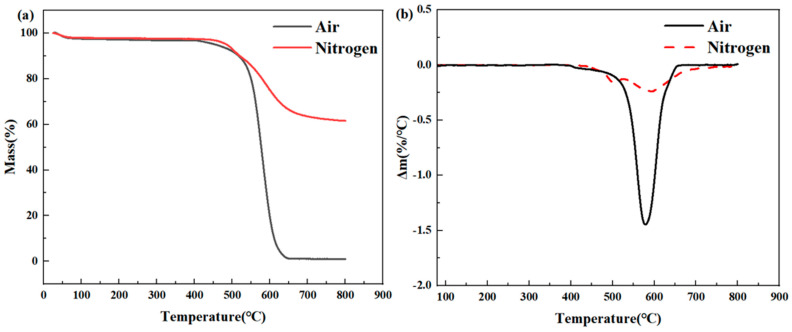
(**a**) TGA curve and (**b**) DTG curve of PMR350 resin.

Firstly, thermogravimetric analysis (TGA) was used to evaluate the thermal stability of phenylienyl-capped polyimide resin (PMR350) in nitrogen and air. Generally, the higher the thermal decomposition temperature, the better the high-temperature resistance of the polymer. The TGA curve is shown in Figure 2, and the specific data are listed in Table 2. It can be observed that PMR350 polyimide resin has only one main thermal degradation stage in nitrogen and air, with no significant thermal degradation weight loss below 500 °C, indicating good heat resistance. This is attributed to the use of phosphoenolpyruvate (PEP) as the end-sealing agent in this polyimide resin. By using 3,4’-Biphenyltetracarboxylic dianhydride (BPDA) as the monomer, the resin was crosslinked to form conjugated macromolecules, resulting in a thermal decomposition temperature of the crosslinked polymer molecules in air higher than 500 °C. At temperatures exceeding 500 °C, the thermal degradation rate in nitrogen and air atmospheres shows a significant difference, with the thermal stability in a nitrogen atmosphere notably superior to that in an air atmosphere. In a nitrogen atmosphere, the 5% thermal decomposition temperature (T_d5%_) of the resin is 485 °C, the maximum decomposition rate temperature is 598 °C, and the residual mass (CR800) at 800 °C is 61.63%. However, in an air atmosphere, the T_d5%_ of the resin is 456 °C, a decrease of 29 °C, and the maximum weight loss rate temperature decreases slightly. Beyond 578 °C, the weight loss rate increases sharply until reaching approximately 660 °C. The resin’s decomposition rate decreases gradually, and the residual mass CR800 value is approximately 0. These results indicate that the thermal degradation rate of PMR350 polyimide resin in a nitrogen atmosphere is lower, resulting in better thermal stability. This is because, in the absence of external factors, the weight loss during resin degradation is primarily due to the breakage of the molecular chain. In contrast, in an air atmosphere, oxygen accelerates and promotes the thermal degradation of polyimide resin. Under an air atmosphere, the polyimide molecular chain degrades more thoroughly, leading to a higher weight loss rate and greater performance loss of the resin. For the actual operating conditions of aviation and space vehicles, the presence of air is essential. Therefore, it is crucial to study the thermal oxidation stability of polyimide resin in high-temperature air environments.

### 3.3. Thermal Oxidation Stability

**Figure 3:**
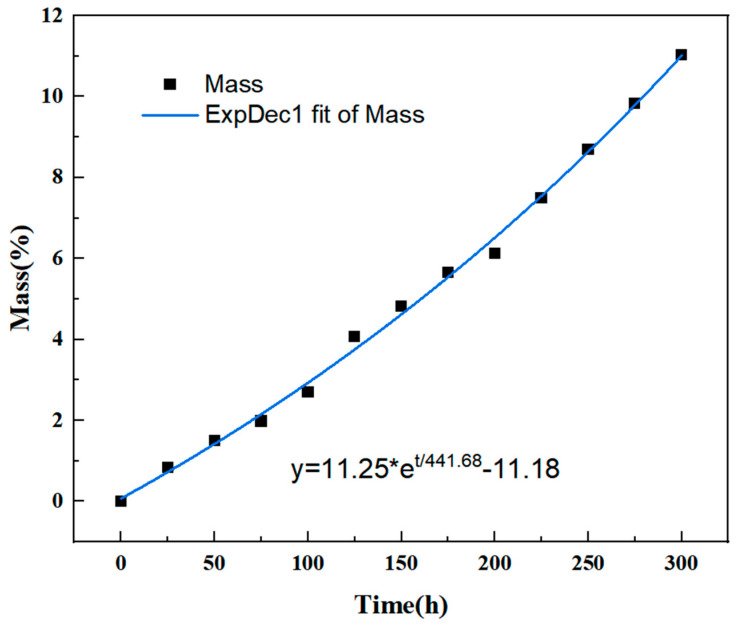
Isothermal aging weight loss curve and linear fitting curve of PMR350 resin.

In order to accurately analyze the thermal oxidation stability of phenylethinyl-capped polyimide resin in a long-term working environment, a target service temperature of 350 °C was selected as the test temperature point. The change in weight loss rate during the isothermal aging process at 350 °C for 300 h was tested using the isothermal oxygen aging method. Mass changes were measured every 25 h, and the weight loss rate at each time point of thermal assessment was calculated. The data on the thermal oxidation weight loss rate at specific time points are presented in Table 3 and Figure 3 below.

As shown in Figure 3, the results indicate that the weight loss rate of polyimide resin gradually increases with the aging time extension. The fit of the thermal weight loss curve is approximately consistent with the first-order exponential growth function $y=-11.18+11.25e^{t/441.68}$; in the formula, ‘t’ represents time, and ‘y’ represents the weight loss rate. The thermal weight loss curve of PMR350 resin during thermal assessment at 350 °C demonstrates a strong agreement with the first-order exponential growth function, implying that the thermal oxidation weight loss rate of PMR350 resin rises with the duration of thermal assessment.

**Figure 4:**
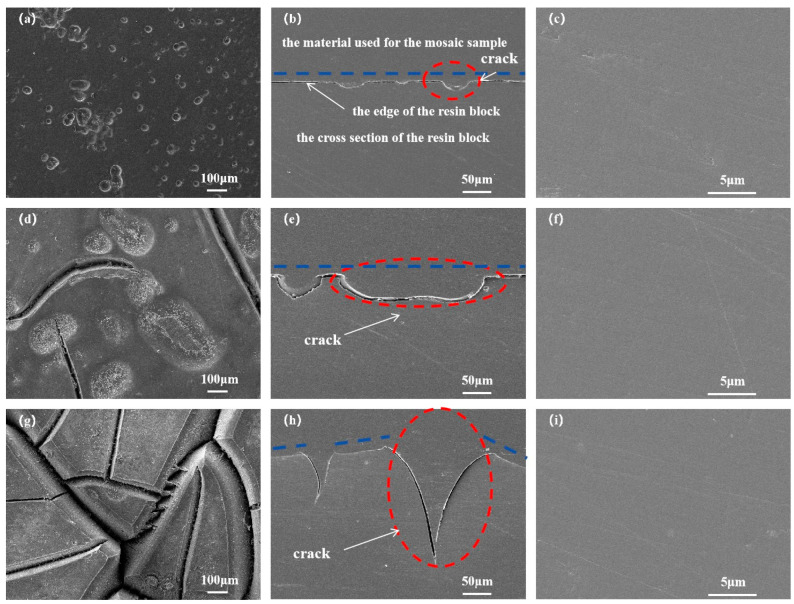
PMR350 resin: (**a**) 100 h surface; (**b**) 100 h section edge position; (**c**) 100 h section center position; (**d**) 200 h surface; (**e**) 200 h section edge position; (**f**) 200 h section center position; (**g**) 300 h surface; (**h**) 300 h section edge position; (**i**) 300 h SEM image of central position of section.

In order to explain the change in the weight loss rate of phenylidene-terminated polyimide resin after aging at 350 °C for 25, 50, 100, 200, and 300 h, the surface and cross-section morphologies of the resin were observed using scanning electron microscopy. The result is shown in Figure 4, in which the blue line refers to the change in the flatness of the resin edges and the red circle indicates the crack observed after aging. It was observed that small pores of varying sizes and depths were distributed on the surface of the aged resin, as depicted in Figure 4a. These pores were a result of the oxidative degradation of the resin, and the number of voids increased gradually with prolonged aging time. The depth of the voids also increased progressively with the duration of aging, as shown in Figure 4a,d,g. After 200 h of thermal testing, the resin surface exhibited a groove, and after 300 h, the depth of the groove significantly increased. In Figure 4h, it is evident that the edge of the resin block lost its parallelism, indicating bending deformation due to long-term thermal aging. By comparing the surface and cross-section topographies of the aged resin, it was observed that, although the surface of the resin had densely distributed pores, there were almost no visible pores inside the resin. This suggests that oxidation decomposition primarily occurs in the oxygen-enriched surface region, while degradation in the oxygen-poor internal area is minimal. This indicates that, besides matrix decomposition caused by thermal fracture of the polymer molecular chain, oxidative decomposition is another significant mechanism of resin degradation. This reaction is more pronounced and rapid on the surface of oxygen-rich resin materials, consistent with the results of TGA. As aging time increased, the pores resulting from resin degradation multiplied, leading to a larger interaction area between oxygen and resin. The accelerated resin oxidation and degradation over time explain the resin weight loss rate trend in Figure 3, which follows a first-order exponential growth function (ExpDec1), signifying gradual acceleration with increasing temperature exposure.

**Figure 5:**
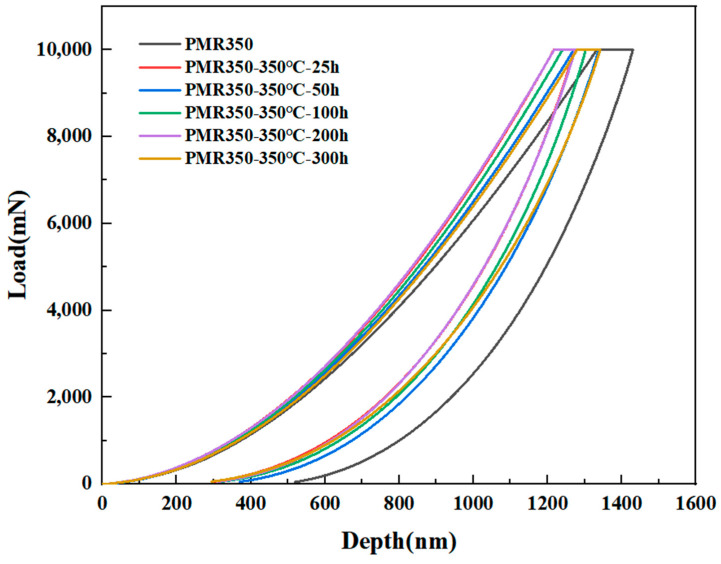
Nanoindentation curves of PMR350 resin before and after thermal assessment.

In addition, the micromechanical properties of PMR350 resin before and after thermal aging were measured using nanoindentation. Based on the SEM image of the sample cross-section, the surface of the resin block was significantly eroded by oxygen, while the interior showed no significant oxidation. Therefore, the edge of the resin was chosen for the nanoindentation experiment. The results of the hardness and modulus tests are presented in Table 4 below, and the nanoindentation curve is depicted in Figure 5.

Due to the post-curing of the resin at 350 °C, the hardness of the resin after a 25 h thermal test is slightly greater than that of the resin without a thermal test. Considering the measurement error, the hardness of the resin after thermal testing for different durations is basically maintained at approximately 0.48 GPa, with slight fluctuations. This indicates that, despite the contact surface between the resin and oxygen being eroded to varying degrees after prolonged thermal aging, the hardness of the remaining resin still exhibits a high retention rate. This demonstrates that the PMR350 resin possesses good thermal oxidation stability.

### 3.4. Thermal Oxidation Degradation Kinetics

**Figure 6:**
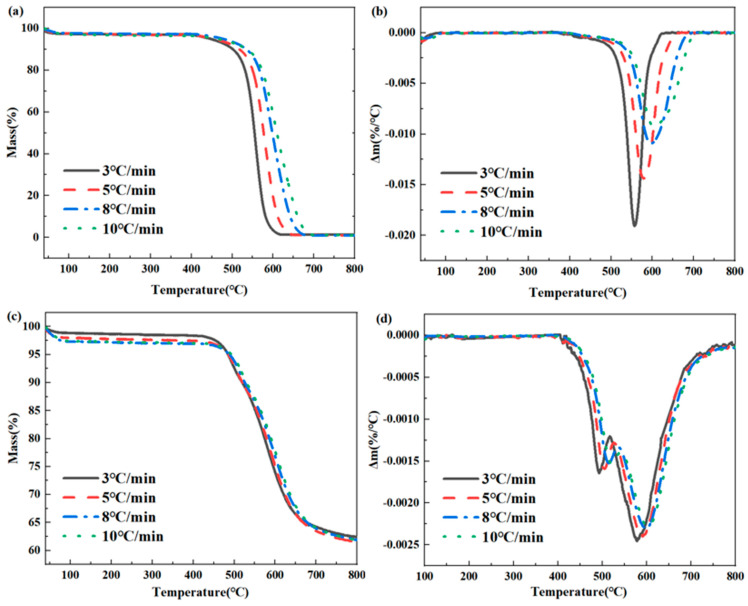
Thermal degradation of PMR350 resin under different heating rates in air atmosphere (**a**) TGA, (**b**) DTG, thermal degradation of PMR350 resin (**c**) TGA, (**d**) DTG in nitrogen atmosphere.

In order to further explore the thermal oxidation degradation mechanism of the phenylacetylene group, the degradation dynamics were studied through non-isothermal weight loss (TGA). The resin samples were heated from room temperature to 800 °C at heating rates of 3, 5, 8, and 10 °C/min. The heat loss under air and nitrogen atmospheres was tested, and the corresponding TGA and DTG curves were obtained. The TGA curves of the polyimide film and the corresponding DTG curves are shown in Figure 6. From the figure, it can be observed that the shape of the TGA curve remains unaffected by the heating rate, indicating that the kinetic process of thermal oxidation degradation is independent of the heating rate. In the air atmosphere, only one peak is present in the DTG curve at different heating rates, suggesting that the thermal oxidative degradation process involves only one main reaction. Conversely, in the nitrogen atmosphere, two peaks are observed in the DTG curve at different heating rates, indicating that the thermal oxidative degradation process involves two main reactions.

#### 3.4.1. Calculating Eas by the Flynn–Wall–Ozawa Method

**Figure 7:**
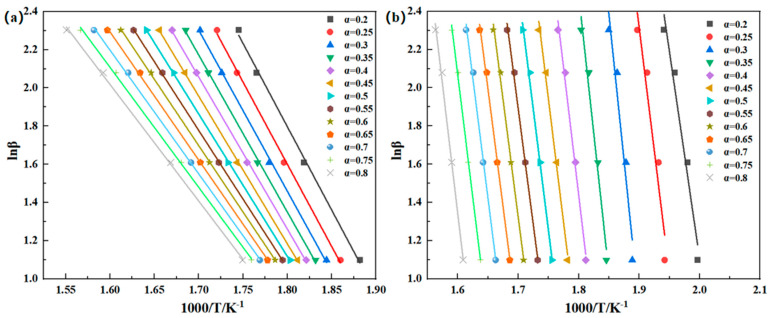
Linear fit of the lnβ − 1000/T curve in (**a**) air and (**b**) nitrogen based on the Flynn–Wall–Ozawa method.

The Flynn–Wall–Ozawa method is a method for measuring Ea values without knowledge of the reaction mechanism. By integrating the basic Equation (2) of the kinetic parameters determined from the TGA data and using the Doyle approximation, we can obtain Equation (3) [27,28]:

(2) $$\frac{\mathrm{dT}}{\mathrm{dt}}=\frac{A}{\beta }\exp(-\frac{E_{a}}{RT})f(\alpha )$$

(3) $$\ln\beta =\ln\frac{AE_{a}}{g\left(\alpha \right)R}-5.3308-\frac{1.052E_{a}}{RT}$$

where g(α) is the integral form of the reaction kinetic function and the expressions of f(α) and g(α) differ in various reaction mechanisms. The activation energy Ea can be determined from the linear relationship between lnβ and 1/T, lnβ to 1000/T, and then linearly fitted. The activation energy of thermal degradation can be obtained from the slope. Figure 7 illustrates the linear fitting curve of lnβ − 1000/T. The apparent mean activation energy of the thermal degradation reaction of PMR350 in the air atmosphere is 61.95 kJ/mol, the mean value of the first main reaction is 248.51 kJ/mol, and the mean value of the second main reaction is 203.64 kJ/mol (as shown in Table 5).

#### 3.4.2. Calculating Eas by the Kissinger–Akahira–Sunose Method

The Kissinger–Akahira–Sunose method is an equal conversion method, as like the Flynn–Wall–Ozawa method. Its expression is shown in Equation (4) [29,30]:

(4) $$\ln\left(\frac{\beta }{T^{2}}\right)=\ln\left[\frac{A\cdot E_{a}}{g\left(\alpha \right)R}\right]-\frac{E_{a}}{RT}$$

**Figure 8:**
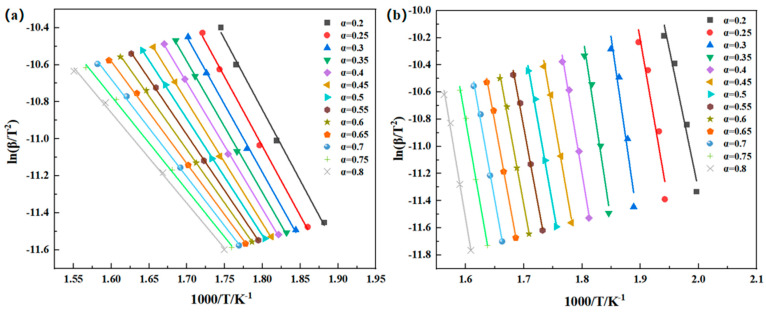
ln(β/T^2^) − 1000/T linear fitting curve in (**a**) air and (**b**) nitrogen based on the Kissinger–Akahira–Sunose method.

As can be observed in Formula (4), when the degree of decomposition (α) is fixed to a specific value, the relationship between ln(β/T^2^) and 1/T is linear for different heating rates. The activation energy of the thermal degradation can be determined from the slope of the straight line. Various α values can be chosen to calculate the activation energy of thermal decomposition at different decomposition levels. By plotting ln(β/T^2^) against 1000/T and performing linear regression, the activation energy of thermal degradation can be calculated from the slope. Figure 8 illustrates the linear regression curve of ln(β/T^2^) − 1000/T, revealing that the apparent mean activation energy of the PMR350 thermal degradation reaction under an air atmosphere is 52.30 kJ/mol. The average value for the first main reaction is 239.51 kJ/mol, and for the second main reaction, it is 193.77 kJ/mol (as indicated in Table 6).

#### 3.4.3. Calculating Eas by the Friedman Method

The Friedman method is a differential method commonly used for determining the kinetic parameters of thermal decomposition reactions. This method necessitates a minimum of four thermal weight loss curves at various heating rates, and the mathematical expression is presented in Equation (5) [31,32]:

(5) $$\ln\left(\frac{d\alpha }{dt}\right)=\ln\left(\beta \frac{d\alpha }{dT}\right)=\ln\left[A\cdot f\left(\alpha \right)\right]-\frac{E}{RT}$$

As can be observed in Formula (4), when the degree of decomposition (α) is fixed to a specific value, the relationship between ln(βdα/dT) and 1/T is linear for different heating rates. The activation energy of the thermal degradation can be determined from the slope of the straight line. Various α values can be chosen to calculate the activation energy of thermal decomposition at different decomposition levels. Plot ln(βdα/dT) against 1000/T and perform linear regression to calculate the activation energy of thermal degradation using the slope. Figure 8 illustrates the linear regression curve of ln(βdα/dT) − 1000/T, revealing that the apparent mean activation energy of the PMR350 thermal degradation reaction under an air atmosphere is 57.12 kJ/mol. The average value for the first main reaction is 244.57 kJ/mol, and for the second main reaction, it is 198.45 kJ/mol (as indicated in Table 7).

**Figure 9:**
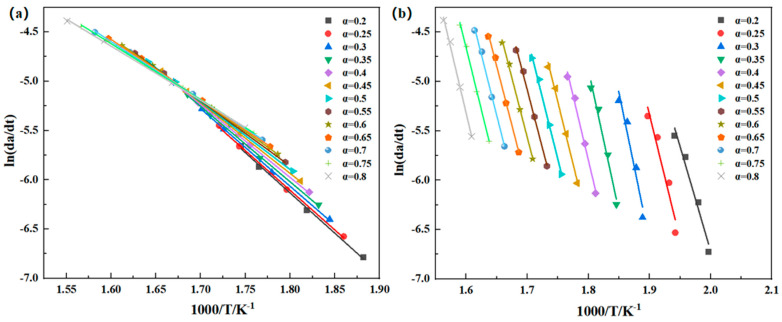
ln(βdα/dT) − 1000/T linear fitting curve in (**a**) air and (**b**) nitrogen based on the Friedman method.

**Figure 10:**
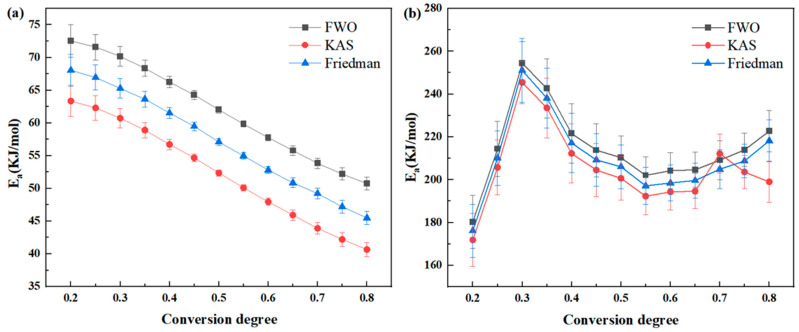
Relationship between Ea and the conversion rate of polyimide degradation in (**a**) air and (**b**) nitrogen.

The values of F–W–O, K–A–S, and Friedman are closely aligned, indicating the reliability of the three methods. The fitted lines in Figure 7, Figure 8 and Figure 9 are not entirely parallel, and the slope of the lines varies with the conversion rate α. This suggests different reaction mechanisms in the PMR resin during the thermal decomposition process. The relationship between Ea and the conversion rate α is shown in Figure 10. Under an air atmosphere, the activation energy of PMR350 resin decomposition gradually decreases with an increase in α, indicating that the thermal decomposition reaction of PMR350 resin in this environment involves at least one phase.

Under a nitrogen atmosphere, the activation energies within 0.3 ≤ α ≤ 0.35 and 0.55 ≤ α ≤ 0.65 are approximately 240 kJ/mol and 200 kJ/mol, respectively. This suggests that the thermal oxidative degradation process of polyimide involves two primary reactions, with the activation energy for each reaction in a nitrogen atmosphere being significantly higher than that in an air atmosphere. When PMR350 resin and its composite materials are in actual service, they inevitably come into contact with oxygen. Under certain extreme conditions, they may even be exposed to a thermal oxygen environment for extended periods. Given that the presence of oxygen greatly accelerates the degradation of PMR350 resin, enhancing the long-term thermal and oxidative aging resistance of the matrix resin remains a key focus for future research.

### 3.5. Determination of the Reaction Model

To infer the thermal degradation mechanism of resin, researchers often employ the Coats–Redfern method to establish a linear relationship by utilizing characteristic values at different conversion rates (α) to calculate the activation energy. The Coats–Redfern method is typically applied using a simplified formula, as shown in Equation (6). Depending on the various degradation processes, the corresponding theoretical function g(α) is presented in Table 8 [26]. The activation energy (Ea) can be determined from the slope of the ln[g(α)/T²] − 1/T diagram. The activation energies of the different degradation processes are then compared with the results in Section 3.3 to elucidate the reaction mechanism.

(6) $$\ln\left(\frac{g\left(\alpha \right)}{T^{2}}\right)=\ln\left[\frac{A\cdot R}{\beta E_{a}}\right]-\frac{E_{a}}{RT}$$

**Figure 11:**
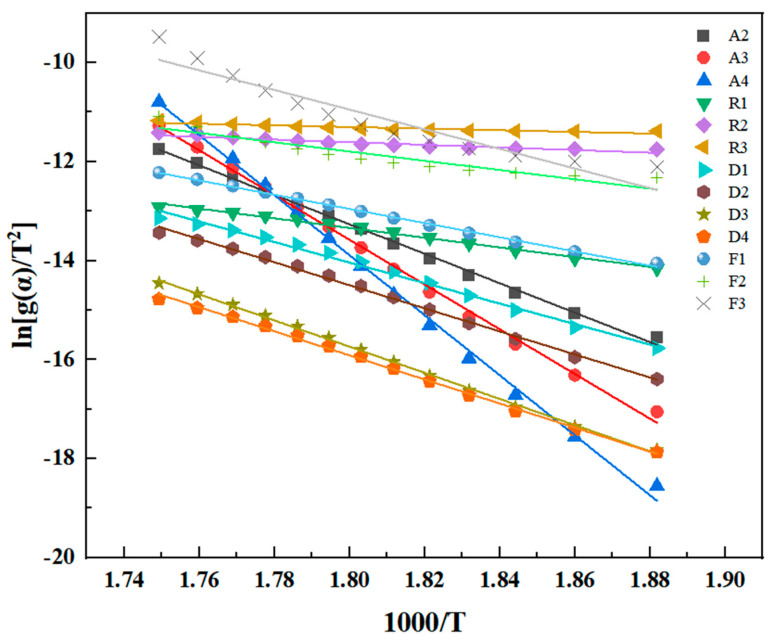
Linear fitting curve of the activation energy of the reaction according to the Coats–Redfern method under an air atmosphere.

Figure 11 illustrates the linear fitting curve of the activation energy for various degradation processes of PMR350 resin in an air atmosphere, as determined by the Coats–Redfern method. The specific parameters are detailed in Table 9. The activation energy for the thermal degradation of PMR350 resin in an air atmosphere is approximately 57 kJ/mol, which is most comparable to the activation energy of the F_2_ reaction. The solid thermal degradation process of PMR350 follows a random nucleation with two nuclei on the individual particle (F_2_), represented by the integral form g(α) = 1/(1 − α).

**Figure 12:**
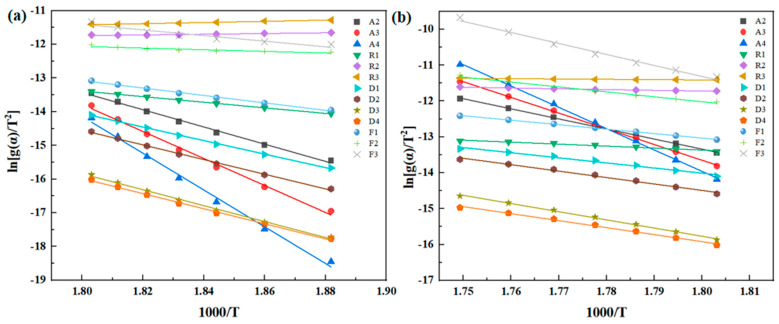
Linear fitting curve: (**a**) Process 1 and (**b**) Process 2 of reaction activation energy according to Coats–Redfern method.

Figure 12 illustrates the linear fitting curve of the activation energy for the reaction under a nitrogen atmosphere, as determined by the Coats–Redfern method. The specific parameters are detailed in Table 10. According to 3.4, the solution of PMR350 in nitrogen is divided into two distinct processes. To enhance the accuracy of the fitting, the analysis is conducted in these two processes. The activation energy values obtained under a nitrogen atmosphere, as described in Section 3.4, fall within the reaction degree ranges of 0.3 to 0.35 and 0.55 to 0.65, corresponding to approximately 240 kJ/mol and 200 kJ/mol, respectively. When compared with the activation energies of various reactions, the first thermal degradation process closely aligns with the A2 calculations, while the second thermal degradation process is most similar to the D2 calculations. The initial phase of solid thermal degradation is governed by the nucleation and growth process (A_2_), represented by the integral form g(α) = [−ln(1 − α)]^2^. This indicates that the thermal oxidative degradation of PMR350 polyimide initiates at defects caused by high temperatures and subsequently propagates throughout the material; a similar result is also reported in previous research [33]. The second process adheres to a three-dimensional diffusion model (D3), with its integral form expressed as g(α) = [1 − (1 − α)^1/3^]^2^.

## 4. Conclusions

(1) The thermal degradation performance of PMR350 under various atmospheres was investigated using TGA. The results indicated that PMR350 exhibited a higher Td5% in a nitrogen atmosphere, which was 29 °C higher than that in air. Additionally, PMR350 demonstrated improved residual weight and a lower thermal degradation rate, suggesting that oxygen accelerated and promoted thermal degradation.
(2) The thermal oxidation stability of polyimide resin was assessed using the isothermal oxidative aging method, which revealed that the weight loss rate during isothermal oxidative aging followed a first-order exponential growth function and accelerated with increasing aging time. The microscopic mechanical properties of the PMR350 before and after aging were characterized, and the hardness of the resin exhibited a high retention rate, demonstrating optimized thermal oxidation stability.
(3) The thermal degradation dynamics were studied through non-isothermal weight loss (TGA). The degradation and activation energy are calculated using the Flynn–Wall–Ozawa method in non-isothermal dynamics, Kissinger–Akahira–Sunose method, and Friedman method. The calculation results indicate that the thermal decomposition reaction of PMR350 resin in an air atmosphere consists of at least one stage. The reaction model g(α) conforms to the F2 model, with the integral form expressed as g(α) = 1/(1 − α). In contrast, the thermal decomposition reaction in a nitrogen atmosphere involves at least two stages, with the reaction models g(α) conforming to the A2 and D3 models, respectively. The integral forms are g(α) = [−ln(1 − α)]^2^ and g(α) = [1 − (1 − α)^1/3^]^2^. This research content offers a theoretical foundation for predicting the thermal weight loss behavior of polyimide resin in various high-temperature oxidation environments. It also provides essential data to support its long-term engineering applications in extreme aerospace conditions.

## Figures and Tables

**Table 1:** Factory parameters of PMR350 resin.

Brand	Manufacturer	Solvent	Solid Content/%	Viscosity/mPa·s
PMR350	Ningbo Institute of Materials Technology and Engineering, Chinese Academy of Sciences	ethanol	47	187

**Table 2:** Decomposition temperature, maximum decomposition rate temperature, and residual mass of PMR350 polyimide resin under air and nitrogen atmospheres.

Test Atmosphere	T_d5%_/°C	T_dmax_/°C	CR800/%	Δm_max_/(%/°C)
Nitrogen	485	598	61.53	0.0025
Air	456	578	0	0.0191

**Table 3:** Weight loss/% of PMR350 resin cured at 350 °C isothermal aging (air).

Thermal Aging Time/h	25	50	100	200	300
Thermogravimetric ratio/%	0.83	1.49	2.70	6.13	11.03

**Table 4:** Hardness and modulus of PMR350 resin before and after thermal examination.

Sample	Hardness/GPa	Modulus/GPa
PMR350	0.36 ± 0.01	3.88 ± 0.14
PMR350-350 °C-25 h	0.50 ± 0.03	4.54 ± 0.01
PMR350-350 °C-50 h	0.44 ± 0.04	4.14 ± 0.01
PMR350-350 °C-100 h	0.48 ± 0.09	4.38 ± 0.03
PMR350-350 °C-200 h	0.50 ± 0.07	4.50 ± 0.02
PMR350-350 °C-300 h	0.46 ± 0.07	3.94 ± 0.02

**Table 5:** The thermal degradation activation energy of PMR350 resin in the air and nitrogen atmospheres calculated based on the Flynn–Wall–Ozawa method.

α	Air		Nitrogen	
	Ea/KJ·mol^−1^	R^2^	Ea/KJ·mol^−1^	R^2^
0.2	72.5 ± 2.7	0.99768	180.3 ± 12.4	0.97013
0.25	71.6 ± 2.0	0.99852	214.4 ± 12.8	0.94156
0.3	70.2 ± 1.5	0.99904	254.4 ± 10.0	0.95291
0.35	68.4 ± 1.2	0.99936	242.7 ± 13.9	0.98099
0.4	66.3 ± 0.9	0.99964	221.6 ± 13.8	0.99227
0.45	64.3 ± 0.7	0.99978	213.8 ± 12.4	0.99339
0.5	62.0 ± 0.5	0.99986	210.3 ± 10.2	0.99529
0.55	59.8 ± 0.5	0.99988	202.0 ± 8.7	0.99632
0.6	57.8 ± 0.6	0.99986	204.2 ± 8.5	0.99658
0.65	55.8 ± 0.7	0.99966	204.7 ± 8.1	0.99684
0.7	53.8 ± 0.8	0.99956	209.1 ± 9.0	0.99628
0.75	52.2 ± 0.9	0.99936	214.0 ± 7.8	0.99734
0.8	50.7 ± 1.0	0.99926	222.7 ± 9.6	0.99626
Average: 62.0 ± 7.3			First step: 248.5 ± 5.9	
			Second step: 203.6 ± 1.1	

**Table 6:** The thermal degradation activation energy of PMR350 resin under air and nitrogen atmospheres calculated based on the Kissinger–Akahira–Sunose method.

α	Air		Nitrogen	
	Ea/KJ·mol^−1^	R^2^	Ea/KJ·mol^−1^	R^2^
0.2	63.4 ± 2.4	0.99712	171.9 ± 12.4	0.96715
0.25	62.3 ± 2.9	0.99818	205.8 ± 12.8	0.93679
0.3	60.7 ± 1.5	0.99884	245.5 ± 10.0	0.94957
0.35	58.9 ± 1.1	0.99924	233.5 ± 13.9	0.97949
0.4	56.7 ± 0.8	0.99958	212.3 ± 13.9	0.99156
0.45	54.7 ± 0.6	0.99976	204.5 ± 12.4	0.99273
0.5	52.4 ± 0.5	0.99984	200.7 ± 10.3	0.99481
0.55	50.1 ± 0.5	0.99982	192.3 ± 8.7	0.99592
0.6	48.0 ± 0.5	0.99974	194.3 ± 8.5	0.99620
0.65	45.9 ± 0.8	0.99940	194.7 ± 8.2	0.99650
0.7	43.9 ± 0.9	0.99918	212.2 ± 9.0	0.99586
0.75	42.2 ± 1.0	0.99880	203.6 ± 7.8	0.99704
0.8	40.7 ± 1.1	0.99860	199.0 ± 9.7	0.99586
Average: 52.3 ± 7.5			First step: 239.5 ± 6.0	
			Second step: 193.8 ± 1.0	

**Table 7:** The thermal degradation activation energy of PMR350 resin in air and nitrogen atmospheres calculated based on the Kissinger–Akahira–Sunose method.

α	Air		Nitrogen	
	Ea/KJ·mol^−1^	R^2^	Ea/KJ·mol^−1^	R^2^
0.2	68.1 ± 2.4	0.99742	176.2 ± 13.4	0.96869
0.25	66.9 ± 1.9	0.99836	210.1 ± 12.8	0.93923
0.3	65.3 ± 1.5	0.99854	251.1 ± 14.0	0.95129
0.35	63.6 ± 1.2	0.99932	238.1 ± 13.9	0.98026
0.4	61.5 ± 0.9	0.99962	217.1 ± 13.8	0.99194
0.45	59.5 ± 0.6	0.99978	209.3 ± 12.4	0.99307
0.5	57.1 ± 0.5	0.99986	206.1 ± 10.2	0.99507
0.55	55.0 ± 0.5	0.99986	197.2 ± 8.7	0.99612
0.6	52.8 ± 0.5	0.99980	198.5 ± 8.5	0.99640
0.65	50.9 ± 0.8	0.99956	199.7 ± 8.2	0.99668
0.7	49.2 ± 0.9	0.99940	204.9 ± 9.0	0.99608
0.75	47.2 ± 1.0	0.99914	208.8 ± 7.8	0.99720
0.8	45.5 ± 1.0	0.99900	218.2 ± 9.7	0.99608
Average: 57.1 ± 7.4			First step: 244.6 ± 6.5	
			Second step: 198.5 ± 1.0	

**Table 8:** Algebra expression formula [26] for the solid-state thermal degradation process of the resin [26].

Symbol	g(α)	Solid-State Processes
A_2_	[−ln(1 − α)]^2^	Nucleation and growth (Avrami equation 1)
A_3_	[−ln(1 − α)]^3^	Nucleation and growth (Avrami equation 2)
A_4_	[−ln(1 − α)]^4^	Nucleation and growth (Avrami equation 3)
R_1_	α	Phase boundary controlled reaction (one-dimensional movement)
R_2_	2[1 − ln(1 − α)^1/2^]	Phase boundary controlled reaction (contracting area)
R_3_	3[1 − ln(1 − α)^1/3^]	Phase boundary controlled reaction (contracting volume)
D_1_	α^2^	One-dimensional diffusion
D_2_	(1 − α)ln(1 − α) + α	Two-dimensional diffusion (Valensi equation)
D_3_	[1 − (1 − α)^1/3^]^2^	Three-dimensional diffusion (Jander equation)
D_4_	[1 − (2/3)α] − (1 − α)^2/3^	Three-dimensional diffusion (Ginstling–Brounshtein equation)
F_1_	−ln(1 − α)	Random nucleation with one nucleus on the individual particle
F_2_	1/(1 − α)	Random nucleation with two nuclei on the individual particle
F_3_	1/(1 − α)^2^	Random nucleation with three nuclei on the individual particle

**Table 9:** The specific parameters of the reaction activation energy were solved according to the Coats–Redfern method.

Symbol	Ea/KJ·mol^−1^	R^2^
A_2_	247.6 ± 3.6	0.99873
A_3_	375.9 ± 5.5	0.99875
A_4_	504.3 ± 7.3	0.99877
R_1_	81.6 ± 2.1	0.99581
R_2_	22.1 ± 2.3	0.94847
R_3_	13.8 ± 1.8	0.92192
D_1_	172.3 ± 4.3	0.99628
D_2_	193.8 ± 3.3	0.99816
D_3_	219.9 ± 2.9	0.98867
D_4_	202.4 ± 3.2	0.97088
F_1_	119.2 ± 1.7	0.99864
F_2_	63.9 ± 8.0	0.94672
F_3_	165.0 ± 6.1	0.95191

**Table 10:** The specific parameters of the activation energy of the reaction were solved according to the Coats–Redfern method.

Symbol	Ea_1_/KJ·mol^−1^	R^2^	Ea_2_/KJ·mol^−1^	R^2^
A_2_	243.8 ± 6.9	0.99964	212.6 ± 2.8	0.99735
A_3_	364.2 ± 10.9	0.99967	333.2 ± 4.2	0.99732
A_4_	494.6 ± 14.0	0.99968	453.9 ± 5.6	0.99730
R_1_	45.7 ± 0.7	0.98749	69.2 ± 3.3	0.99976
R_2_	18.3 ± 1.2	0.99032	8.6 ± 1.2	0.95607
R_3_	7.2 ± 0.9	0.95311	14.5 ± 1.0	0.99128
D_1_	120.8 ± 2.5	0.98505	167.1 ± 6.5	0.99945
D_2_	149.1 ± 3.7	0.99202	191.1 ± 6.0	0.99893
D_3_	189.6 ± 5.3	0.97644	196.6 ± 4.6	0.65543
D_4_	162.4 ± 4.3	0.99914	186.2 ± 5.5	0.98910
F_1_	103.3 ± 3.0	0.99954	91.9 ± 1.4	0.99743
F_2_	114.2 ± 4.3	0.99267	20.4 ± 6.2	0.90289
F_3_	255.5 ± 6.0	0.99413	69.5 ± 7.4	0.95610

## Data Availability

Data are contained within the article.
